# Prognostic and tumor immunity implication of inflammatory bowel disease-associated genes in colorectal cancer

**DOI:** 10.1186/s40001-022-00720-0

**Published:** 2022-06-13

**Authors:** Di Wang, Biao Xie

**Affiliations:** Department of Gastroenterology, People’s Hospital of Longhua, Shenzhen, 518109 People’s Republic of China

**Keywords:** Colorectal cancer, IBD-associated genes, Prognostic signature, Immune cells infiltration, Biomarkers

## Abstract

**Background:**

Epidemiologic studies continue to emphasize that increasing patients with inflammatory bowel disease (IBD) develop to colorectal cancer (CRC). Although the function and mechanisms of IBD-associated genes (IBDGs) in CRC tumorigenesis have been extensively researched, the implications of IBDGs in the prognosis value and tumor immunity of CRC remain unclear.

**Results:**

In this study, the expression, pathological stages and prognostic value of IBDGs in CRC were systematically analyzed, and 7 prognostic genes including CDH1, CCL11, HLA–DRA, NOS2, NAT2, TIMP1 and TP53 were screened through LASSO–Cox regression analysis. Then, a prognostic signature was established based on the 7 prognostic genes, and the model exhibited a good ability in risk stratification of CRC patients. Subsequent results showed that the genetic alterations of the 7 prognostic genes exhibited more significant and extensive influence on immune cells infiltration in colon adenocarcinoma than that in rectal adenocarcinoma. Meanwhile, immune cells infiltration also showed a significant difference between low-risk group and high-risk group. What’s more, 7 prognostic genes-based risk stratification was associated with microsatellite instability, and its prognostic characteristics were significantly negatively correlated with mismatch repair genes.

**Conclusions:**

This study provided a promising insight that the 7 IBDGs could be used as valuable biomarkers for prognostic diagnosis and personalized immunotherapy of CRC patients.

**Supplementary Information:**

The online version contains supplementary material available at 10.1186/s40001-022-00720-0.

## Background

As the world’s top three incidence rate cancer, colorectal cancer (CRC) is also the major reason for cancer-related deaths [1]. Epidemiologic studies have confirmed that colorectal cancer develops from long-term and multistep precancerous lesions. CRC has become a major cause of death in two main inflammatory bowel diseases (IBDs), ulcerative colitis (UC) and Crohn's colitis (CD), and there are about 10 to 15% of IBD patients die from CRC [2]. IBD is an idiopathic disease caused by excessive and chronic inflammation of gastrointestinal tract. The symptoms of IBD are rectal bleeding and abnormal weight loss [3]. Epidemiologic studies indicate that patients with UC or CD are more easily to develop CRC. It’s reported that the risk of CRC in IBD patients is about 2–3 fold higher than that in the general population [4, 5]. Statistically, IBD is responsible for approximately 2% of CRC patients identified each year, and it has ranked as the third commonest disease related to high risk of CRC, familial adenomatous polyposis ranked first and hereditary non polyposis colorectal cancer syndrome ranked second [6].

Chronic or recurrent inflammatory injury of intestinal mucosa has an explicit mechanism in the occurrence of IBD-related CRC [7]. As a risk factor, the influence of IBD on CRC tumorigenesis is closely related to the degree, duration and severity of inflammation [8]. According to statistics, the incidence rates corresponded to cumulative probabilities of IBD-related CRC, 10 years 2%, 20 years 8% and 30 years 18% [9]. Besides, IBD-related CRC patients appear to have greater mortality rate and poorer prognosis compared with patients with CRC without IBD history. The recent data show that the mortality of IBD-related CRC patients increased 1.7-fold compared with the IBD uncorrelated CRC patients. Accumulating researches attribute this problem to difficulties in cure IBD completely and early detection of premalignant lesions [10].

Benefit from both CRC screening and mucositis control, the incidence and mortality of CRC has been decreasing in the past 20 years [11]. However, recent studies have showed a disturbing trend that the incidence rate of young people (< 50 years) is increasing [12]. Notably, IBD-related CRC patients are 7.7 year younger comparing with the uncorrelated at the time of diagnosis, and the average interval from colitis to CRC was 16–21 years [13]. Meanwhile, an increased incidence rate of IBD-related CRC for both CD of 2.64% and UC of 2.75% also has been reported [6]. Thus, it is necessary that further characterization of the implication in evolutionary trajectory between IBD and CRC, which will play an important role in improving clinical testing, risk stratification of molecular biomarkers and chemical prevention of cancer, and provide new opportunities for targeted therapies.

At the molecular level, the chronic and recrudescent character of IBD lead to free radicals release, which induce DNA damage and accumulate mutational genes participated in the carcinogenesis process [14]. The expression of nitric oxide synthase in IBD associated colitis increased, thereby increasing the reactive oxygen and nitrogen species abundance [15, 16]. Comparing with sporadic CRC, this oxidative stress happens earlier in IBD-related CRC, which is related to p53 mutations, MLH1 gene hypermethylation and microsatellite instability [6, 16]. Besides, the occurrence of chronic colitis related tumors may also be related to aberrations in innate and adaptive immune responses. For instance, TNF, an important mediator for mucosal inflammation in IBD, activate NF-κB and upregulate STAT3-mediated antiapoptotic signals through IL-6 in intestinal epithelium [17]. This pathway is important for the mechanism of colon cancerization. Furthermore, there are a large number of infiltrating leukocytes and macrophages in the tumor microenvironment, which are play key roles in chronic inflammation and carcinogenesis [18, 19]. This not only provides an opportunity for the prognosis and predictive biomarkers of CRC, but also provides a potential therapeutic target for anticancer therapy.

IBD-associated genes (IBDGs) as the bridge connect IBD and CRC, their genetic alterations are closely associate with the tumorigenesis and development. Although the function and mechanisms of IBDGs in CRC occurrence have been extensively elucidated, the roles of IBDGs in the prognosis value and tumor immunity of CRC have not yet to be systematically analyzed. Therefore, to clarify the relationship between IBDGs and clinicopathological characteristics in CRC, we use bioinformatics methods to explore potential biomarker associated with tumor immunity, and provide insights for more precise and personalized therapy.

## Methods

### Collection of IBD-associated genes

IBD-associated genes were collected from the MalaCards (https://www.malacards.org/), DisGeNET (https://www.disgenet.org/home/) and Public Health Genomics and Precision Health Knowledge Base (PHGKB, https://phgkb.cdc.gov/PHGKB/phgHome.action?action=home) databases, which provide comprehensive information about human genes and diseases. The term “inflammatory bowel disease” was used as a search keyword. The genes that encode proteins were collected.

### Gene expression, prognosis, pathological stages analysis

Gene expression of CRC tumor and normal tissues, prognosis and pathological stage of CRC patient were analyzed by Gene Expression Profiling Interactive Analysis database (GEPIA, http://gepia.cancer-pku.cn/index.html). GEPIA is an online tool for analyzing RNA-seq. The samples in GEPIA were collected from two public database, the Cancer Genome Atlas (TCGA) and Cancer Genome Atlas and the GenotypeTissue Expression (GTEx) projects. There are 308 tissues (275 tumor and 41 normal) came from TCGA and 307 colon normal tissue from GTEx [20]. Besides, Kaplan–Meier plotter (K–M plotter, http://kmplot.com/analysis/) was used to verified the prognosis result of IBD-associated genes in rectum adenocarcinoma samples. The *p* value < 0.05 was considered as statistically significant difference.

### Analysis of transcription factor binding sites in gene promoter region

The transcription factor binding sites analysis of IBD-associated genes were performed through The Eukaryotic Promoter Database (EPD, https://epd.epfl.ch//index.php). EPD is a database that collected and annotated eukaryotic Pol II promoters, and the transcription initiation sites of these promoters have been confirmed by experiments [21]. Evidence comes from TSS-mapping performed by cap analysis of gene expression (CAGE) or Oligo-capping. To some extent, EPD is helpful to dynamically extract promoter subsets with biological significance for comparative sequence analysis [22]. After search for the specified gene, in the part of “Search Motif Tool”, motif library term chooses “Transcription Factor Motifs (JASPAR CORE 2018 vertebrates)”, and motif term choose the transcription factor, others choose the default Settings. Record the transcription factor binding sites, and visualized by HemI 1.0-Heatmap illustrator software.

### Prognostic signature screening and generation

The overall survival time, overall survival status and RNA-seq data of colorectal cancer patients in TCGA were integrated for LASSO regression analysis. Using the RNA-seq data of the screened genes, each patient was calculated riskscore using the following formula: Riskscore = expression gene 1 ×β gene 1 + ··· + expression gene n × β gene n (β: the regression coefficient derived from LASSO penalized regression).

### Prognostic signature validation and evaluation

Colorectal cancer patients in TCGA were divided into training set and test set according to the ratio of 1:1. Taking the median riskscores of the training set samples as the threshold, the samples were divided into high-risk and low-risk groups. The prediction effect of the prognosis model was evaluated by the survival curve and the time-dependent ROC curve.

### Immune infiltration analysis

The Tumor IMmune Estimation Resource (TIMER, https://cistrome.shinyapps.io/timer/) database was used to analysis the correlation of IBD-associated genes expression with immune infiltrates in CRC tumor, including B cells, CD4 + T cells, CD8 + T cells, neutrophils, macrophages, and dendritic cells, via gene modules. TIMER is an online tool for immune infiltration analysis of different cancers [23]. The data used in TIMER came from TCGA. Using a deconvolution statistical method, TIMER infers the abundance of tumor-infiltrating immune cells (TIICs) from gene expression profiles [24]. By inputting IBD-associated genes selected, scatterplots were output though "gene module”. Scatterplots showed the correlation between the expression and immune infiltration level of IBD-associated genes in CRC.

Infiltrating immune cells of each sample were calculated CIBERSORT algorithm (Cell-type Identification by Estimating Relative Subsets of RNA Transcripts; http://cibersort.stanford.edu). The principle of the CIBERSORT is to identify 22 immune cell subtypes using the patient's gene expression matrix and annotated gene signature expression matrix (LM22). LM22 files were downloaded from CIBERSORT web portal (https://cibersort.stanford.edu/).

### Genomic analysis of IBD-associated genes

The genetic alterations analysis of IBD-associated genes was performed through cBioPortal database (http://www.cbioportal.org/), which includes 636 colorectal adenocarcinoma patients’ 640 tumors samples. cBioPortal is a comprehensive and open-access database, collecting high-quality and multiple types of high-throughput sequencing data for cancer [25]. The parameters were alterations (amplifications, deep deletions, and missense mutations), copy number alterations (CNAs), and mRNA expression z-scores (RNA Seq V2 RSEM), using the default settings. The CNAs came from genomic identification of significant targets in cancer (GISTIC). The genetic alterations of the IBD-associated genes were queried by cBioPortal database which provides a query interface for users.

### Pathway enrichment analysis

The top 100 co-expression genes of each IBD-associated gene in colon adenocarcinoma (COAD) were collected from the GEPIA database. The protein–protein interactional (PPI) network was showed by Cytoscape v3.8.2 software (http://www.cytoscape.org/). Meanwhile, Kyoto Encyclopedia of Genes and Genomes (KEGG) pathway enrichment analysis of the co-expression genes were performed by KEGG using Orthology Based Annotation System (KOBAS) online tool (http://kobas.cbi.pku.edu.cn/genelist/) [26].

GSEA software (version 3.0) were performed to do the gene set enrichment analysis. To evaluate relevant pathways and molecular mechanisms, c2.cp.kegg.v7.4.symbols.gmt were downloaded from Molecular Signatures Database (http://www.gsea-msigdb.org/gsea/downloads.jsp). Based on the gene expression profiles of high-risk and low-risk patients, the minimum gene set was defined as 5, the maximum gene set was defined as 5000, the number of repetitions was set to 1000, and *p* value < 0.05 was considered to be a significantly enriched pathway.

### Establishment of drug gene network

The interaction between drug and gene came from DGIdb (https://dgidb.genome.wustl.edu/) Database. The approved and antineoplastic drugs interacting with prognostic genes were screened. Cytoscape (version 3.82) was used to show the interaction between drugs and genes.

### Statistical data

In this study, all the statistical analysis was conducted in software R version 3.6.0 with relevant packages. Lasso regression analysis was performed by “glmnet” package. Kaplan–Meier’s survival curve was as performed by “survivminer” package. Time-dependent ROC curve analyses were as performed by “survival ROC” package. Univariate, multivariate Cox regression analysis and optimal survival segmentation point were performed by the “survival” package. The immune scores, stromal scores and estimate score of the samples were calculated with the “ ESTIMATE ” package. *T* test was used to compare the differences of various scores and *P* values < 0.05 were considered to be statistically significant. “ggplot2” package was used to draw box diagram and correlation diagram.

## Results

### Differential expression of IBD-associated genes (IBDGs) in CRC patients

**Fig. 1:**
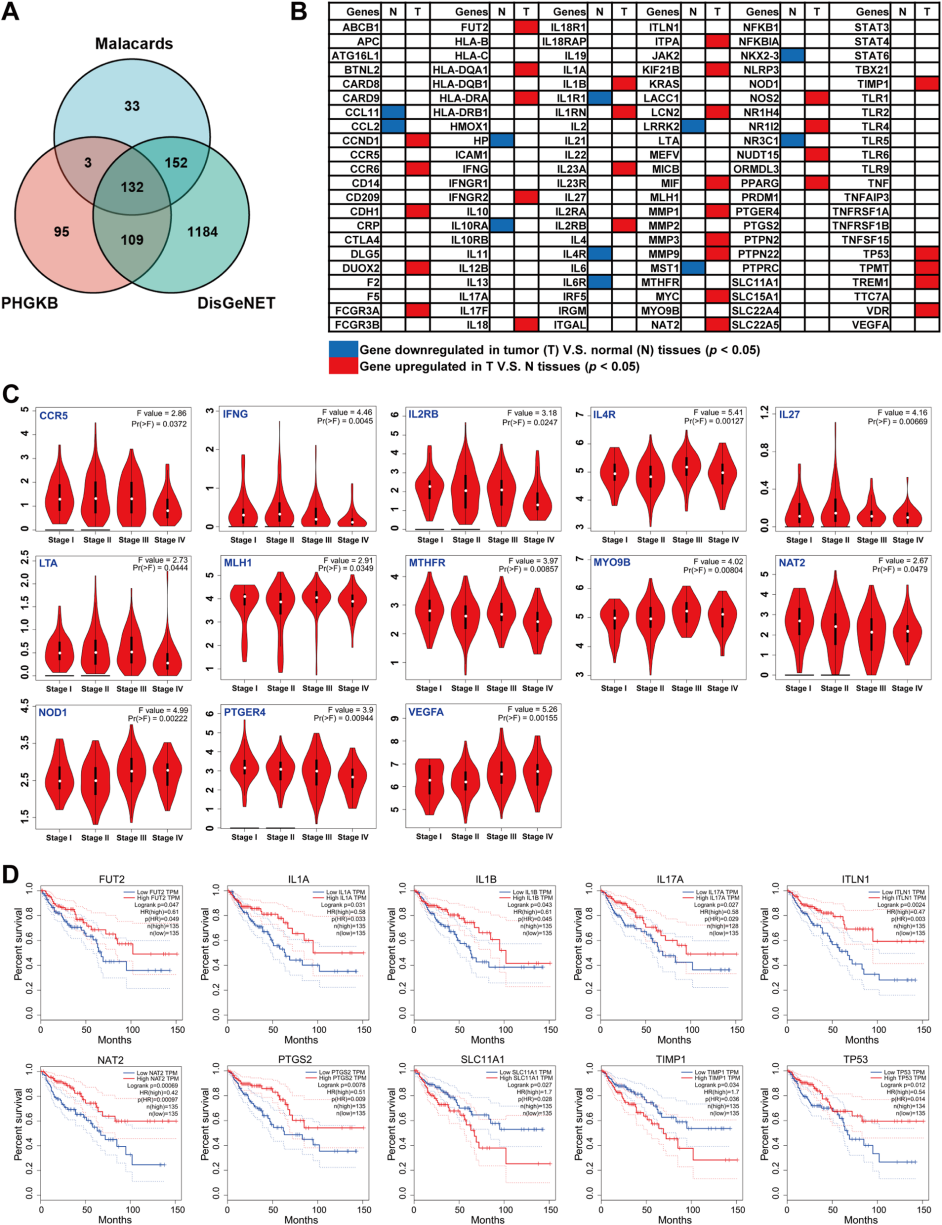
IBDGs influence pathological stages and prognosis of CRC patients. **A** Venn diagram of IBDGs collected from Malacards, PHGKB and DisGeNET databases. **B** Expression of IBDGs in CRC. **C** 13 IBDGs were significant related to the pathological stages of CRC patients. **D** 10 IBDGs have significant effect on the prognosis of CRC patients. The influences of IBDGs on the pathological stages and prognosis of CRC patients were analyzed through GEPIA database

IBD as an important risk factor of CRC has been widely established. To systematically analyze the role of IBD in the occurrence and development of CRC, 320 IBDGs in Malacards database, 339 IBDGs in PHGKB database and 1577 IBDGs in DisGeNET database were collected. We focus on 132 IBDGs, which are the intersection of the three databases (Fig. 1A). Then, the expression of IBDGs in CRC tumor and normal colorectal tissues was analyzed by GEPIA database. The results showed that there were 32 significantly upregulated IBDGs in CRC tissues, include IL1B, IL23A, IL2RB, NOS2, CCND1 and CCR6 (Fig. 1B). Meanwhile, 11 IBDGs mRNA expression were significantly downregulated, include IL10RA, IL1R1, IL4R, IL6R and CCL2.

To further reveal the potential key targets that regulate the expression of IBDGs, we analyzed the transcription factor binding sites in the promoter regions of upregulated and downregulated IBDGs separately, using EPD database. The transcription factors that binding sites in the top 60 were selected, and their mRNA expression in CRC were also analyzed. We found that Ascl2, KLF16, KLF5, TCF3 and TFDP1, all have multiple binding sites in the promoter region of upregulated IBDGs (Additional file 1: Figure S1A). Meanwhile, they are all significantly upregulated in CRC tissues (Additional file 1: Figure S1B). Besides, the results also shown that EBF1, KLF9, MZF1 and ZEB1, all have multiple binding sites in the promoter region of downregulated IBDGs (Additional file 1: Figure S1C), and they are all significantly downregulated in CRC tissues (Additional file 1: Figure S1D). These results demonstrated that the upregulation of Ascl2, KLF16, KLF5, TCF3, TFDP1 and the downregulation of EBF1, KLF9, MZF1, ZEB1 might be responsible for the differential expression of IBDGs, respectively.

### IBDGs affect pathological stages and prognosis of CRC patients

To further evaluate the effect of IBDGs on the pathological process of CRC patients, the pathological stage of CRC patients was analyzed through GEPIA database, first. As Fig. 1C shown that 13 IBDGs exhibited highly variable with the CRC progression. Meanwhile, we could find that CCR5, IFNG, IL2RB, LTA, MTHFR, NAT2 and PTGER4 expression were obviously downregulated, as well as NOD1 and VEGFA expression were clearly upregulated with the CRC progresses. These were suggesting their indicative effect on the pathological stage of CRC patients.

Then, the efficiency of IBDGs in the clinical outcomes of CRC patients were analyzed using GEPIA. The overall survival (OS) curves shown that the high expression of FUT2, IL1A, IL1B, IL17A, ITLN1, NAT2, PTGS2 and TP53 exhibited good prognosis of CRC patients (*p* < 0.05) (Fig. 1D). However, the high expression of SLC11A1 and TIMP1 displayed poor prognosis of CRC patients (*p* < 0.05). Furthermore, the prognostic values of IBDGs in CRC patients were performed by Kaplan–Meier plotter (Additional file 1: Figure S2). We found that CRC patients with high expression of 36 IBDGs, including NAT2, IL2RB, IL4R, IL10RA, NLRP3, NFKB1, et al. have significantly better prognosis. CRC patients with high expression of CD14, IL22, TIMP1, TLR9 and TNFRSF1A have significantly worse prognosis.

### Prognostic signature establishment and verification

**Fig. 2:**
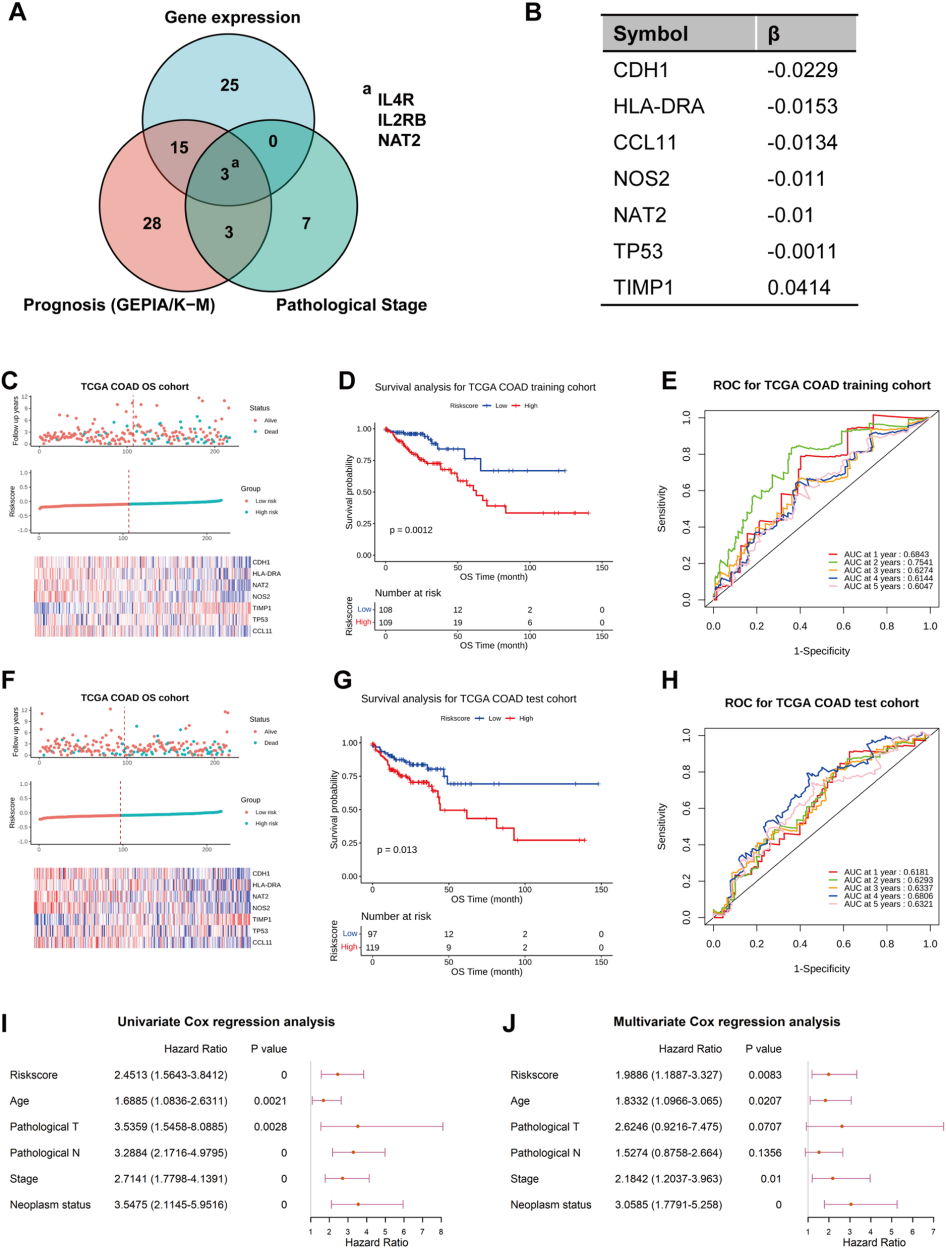
Establishment and verification of prognostic signature. **A** Venn diagram of the IBDGs that differentially expressed in CRC tumor and normal colorectal tissues, and significantly related to pathological stages and prognosis. **B** LASSO–Cox regression coefficient of prognostic signature. **C**, **F** Survival status, riskscores and prognostic signature expression levels of each patient in training and test cohort. **D**, **G** Kaplan–Meier survival curve between high and low riskscore patients in training and test cohort. **E**, **H** Time-dependent (1–5 years) ROC curve comparison of training and test cohort. **I** Univariate and multivariate Cox regression analyses of 7-gene signature and other prognostic factors for OS

Above results we noticed that IL4R, IL2RB and NAT2, which were differentially expressed in CRC tumor, significantly affect both pathological stages and prognosis of CRC patients, collectively (Fig. 2A). Thus, these three genes were included to construct prognostic model, first. After integrating the RNA-seq data of TCGA colorectal cancer patients with clinical prognosis information, a total of 433 patients was collected for prognostic signature establishment and verification. Subsequently, 433 patients were divided into training cohort (217 cases) and test cohort (216 cases). According to the preset riskscore formula, the riskscores of the training cohort patients were calculated, and the patients were divided into low-risk group (108 cases) and high-risk group (109 cases) according to the median value (-0.8393). The riskscore, survival status and the 3 gene expression levels of the two groups of patients are shown in Additional file 1: Figure S3A-C. In training cohort, the survival analysis result showed that the prognosis of the low-risk group was better than that of the high-risk group (Additional file 1: Figure S3B, *p* = 0.041), and time-dependent ROC curve analysis result showed that the AUC of 1–4 years of the prognostic signature were more than 0.6, the AUC of 5 years was less than 0.6 (Additional file 1: Figure S3C). However, in test cohort, although the prognostic signature has the ability to distinguish low-risk and high-risk patients (Additional file 1: Figure S3D, E), ROC curve analysis result showed that the AUC of 1–5 years of the prognostic signature were mostly less than 0.6 (Additional file 1: Figure S3F). These results suggested that the established prognostic signature based on IL4R, IL2RB and NAT2 was not ideal.

Hence, to further construct prognostic signature, we combined the IBDGs that differentially expressed in CRC tumor and normal tissue, and significantly related to pathological stages and prognosis of CRC patients. 7 prognostic genes, including CDH1, CCL11, HLA–DRA, NOS2, NAT2, TIMP1 and TP53, were screened through least absolute shrinkage and selection operator (LASSO) Cox regression analysis (Fig. 2B). According to the above method the patients of training cohort were also divided into low-risk and high-risk groups according to the median value (− 0.0912) (Fig. 2C). The results of survival analysis demonstrated that the prognosis of the low-risk group was significantly better than that of the high-risk group (Fig. 2D, p = 0.0012). Meanwhile, the ROC curve analysis showed that the AUC of 1–5 years of the prognostic signature were more than 0.6 (Fig. 2E). Whereafter, to further verify the reliability of the prognostic model, the riskscores of test cohort and entire cohort were, respectively, divided into low-risk group and high-risk group according to the same threshold − 0.0912 as the training set (Fig. 2F; Additional file 1: Figure S4A). The results showed that survival analysis and ROC curve were consistent with the results of training set (*p* < 0.05, AUC > 0.6) (Fig. 2G, H; Additional file 1: Figure S4B, C). The K–M survival curves revealed a higher survival probability of the low-risk group in this validation cohort. In general, the 7 prognostic genes-based prognostic signature was proven to be valuable in risk stratification.

To evaluate the independence of prognostic signature for predicting overall survival, univariate and multivariate Cox regression analysis were performed to calculate the HRs, 95% CIs, and *p* values. Univariate Cox regression analysis showed that high risk, age, stage, pathological T, pathological N stage and neoplasm status were significantly correlated with overall survival (Fig. 2I, p < 0.05). Multivariate Cox regression analysis showed that except pathological T and pathological N stages, the other variables were independent prognostic factor (Fig. 2J, p < 0.05).

### The 7 prognostic genes are correlated with immune infiltration in CRC

Tumor-infiltrating lymphocytes are an independent predictor of sentinel lymph node status and proliferation and progression of cancers [27, 28]. In this study, the correlation between the 7 prognostic genes (CDH1, CCL11, HLA–DRA, NOS2, NAT2, TIMP1 and TP53) and immune cells infiltration were analyzed. As the results shown in Additional file 1: Table S1, except for TP53, the remaining six IBDGs expression exhibited significant positive correlation with the infiltration of multiple immune cells in colon (COAD) and rectal tumors (READ), such as B cells, CD8 + T cells, CD4 + T cells, macrophages, neutrophils or dendritic cells. Meanwhile, NOS2 expression was significant negative correlation with macrophages in CRC, NAT2 and TIMP1 expression were significant negative correlation with neutrophils in COAD or B cells in READ, respectively.

**Fig. 3:**
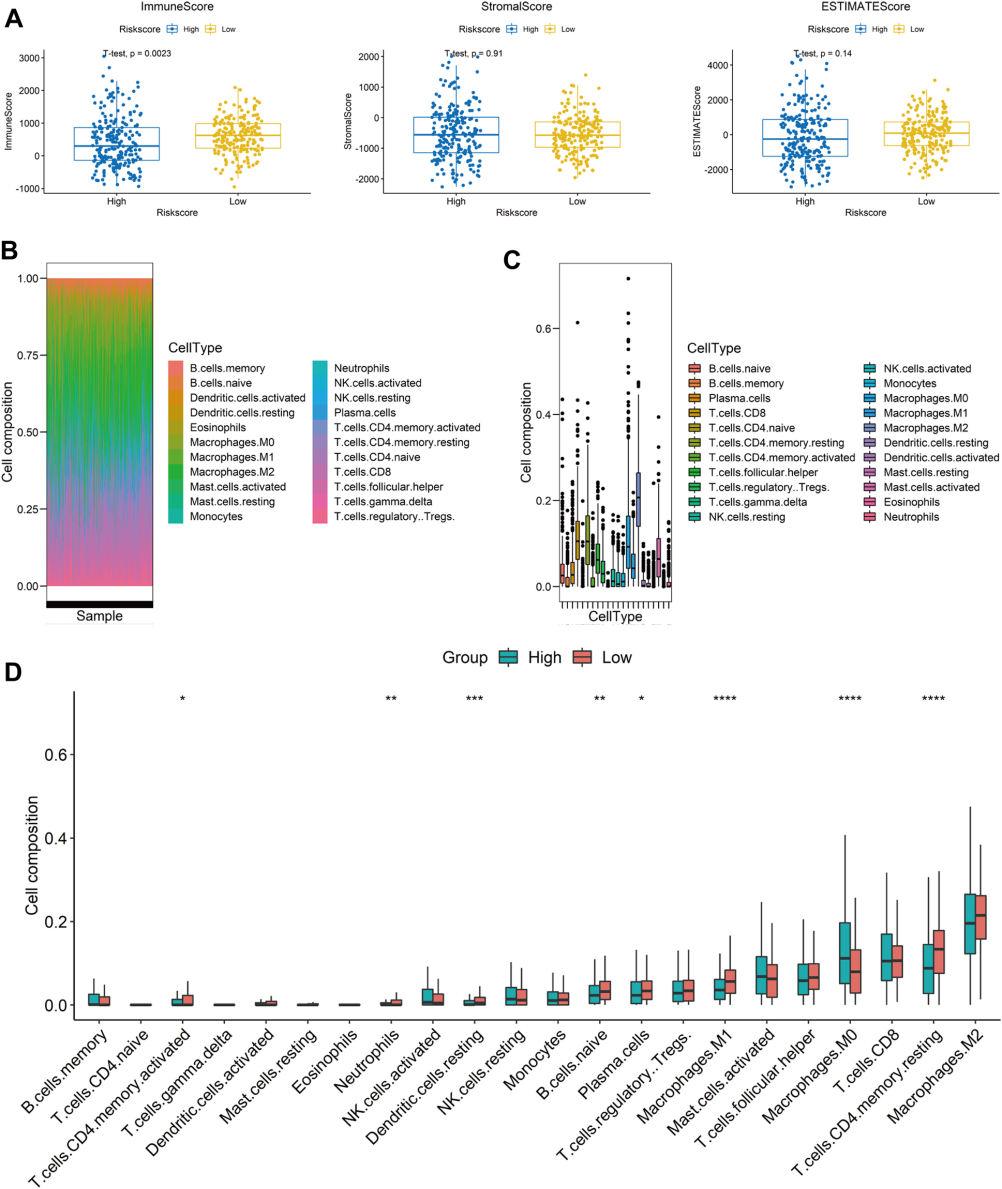
Immune infiltration analysis of 7 prognostic genes-based risk stratification. **A** Comparison of immune score, stromal score and estimate score in high and low risk patients. **B** Composition of 22 immune cells in each CRC patient. **C** Distribution of 22 immune cells in each CRC patient. **D** Comparison of immune cells in high and low risk patients (*P < 0.05, **P < 0.01, ***P < 0.001, ****P < 0.0001)

Furthermore, we evaluated the tumor purity using the ESTIMATE algorithm which can evaluate the proportion of stromal cells and immune cells in malignant tumor tissues through RNA-seq data. The results showed that the immune score of low-risk group was significantly higher than that of high-risk group (*p* = 0.0023), stromal score and ESTIMATE score were have no significant difference in the two group (Fig. 3A). These results indicated that the infiltration of immune cells had a significant difference between low-risk group and high-risk group, although the tumor purity and tumor stroma exhibited no difference in the two group. Thus, we subsequently calculated the infiltration of 22 immune cells in TCGA colorectal cancer patients through ciberport algorithm. As the results shown in Fig. 3B, C, we found that multiple immune cells are highly aberrant, such as macrophages M0, macrophages M2, resetting T cells CD4 memory and T cells CD8. Compared with the low-risk group, the immune infiltration level of macrophages M0 was significantly up-regulated in the high-risk group. Simultaneously, the immune infiltration levels of activated T cells CD4 memory, neutrophils, dendritic cells resting, B cell naïve, plasma cells, macrophages M1 and resting T cells CD4 memory were significantly down-regulated in the high-risk group (Fig. 3D).

### The genetic alterations of the 7 prognostic genes affect CRC immunity

**Fig. 4:**
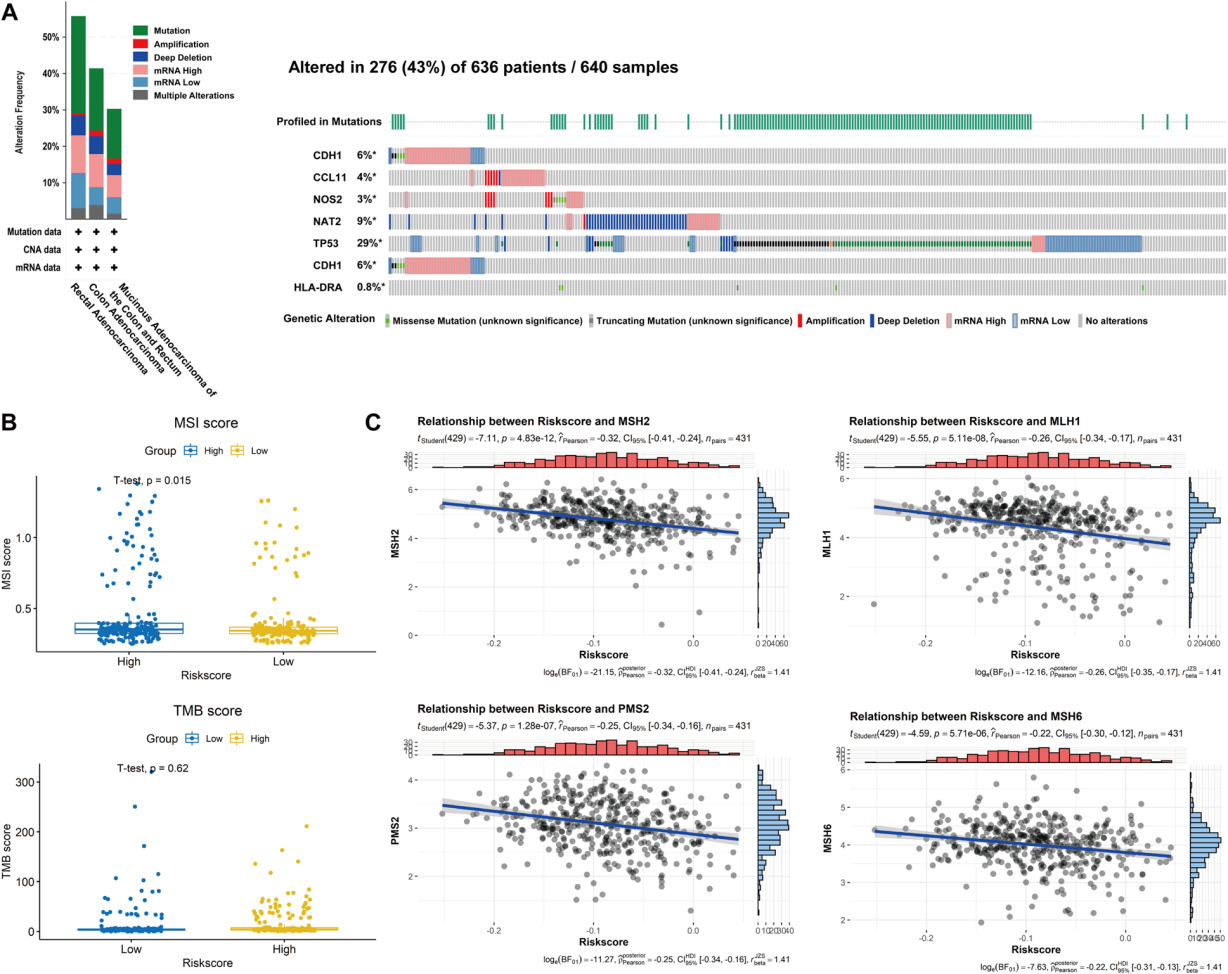
Genetic alterations of the 7 prognostic genes affect microsatellite instability (MSI). **A** Genetic alteration types and frequency (left panel), and genetic alteration profiles (right panel) of three hub genes were analyzed using cBioPortal database. **B** Comparison of MSI score and TMB in high and low risk patients. **C** Pearson correlation analysis of riskscore and mismatch repair genes (MLH1, MSH2, MSH6, and PMS2)

The genetic alterations are closely related to the occurrence and progression of tumors, including tumor uncontrolled proliferation, invasive migration and immune escape [29–31]. Therefore, we first checked the genetic alterations of CDH1, CCL11, HLA–DRA, NOS2, NAT2, TIMP1 and TP53 in CRC patients, in TCGA cohorts via the cBioPortal online tool. The results shown that the 7 genes were altered in 276 (43%) samples of 636 CRC patients. Meanwhile, multiple genetic alterations, especially missense mutation, amplification and deep deletion, were detected in different subtypes of CRC (Fig. 4A). Further analysis using TIMER database, we found that the effect of somatic copy number alterations (SCNA) of the 6 IBDGs (No data related to HLA–DRA was retrieved in the database) on immune infiltration in COAD were more significant and extensive than that in READ, generally (Additional file 1: Figure S5). Particularly, CCL11 gene arm-level gain and high amplification significantly decreased B cell, CD8 + T cell, neutrophil and dendritic cells infiltration in COAD, whereas all the SCNA of CCL11 has nothing effect on immune cells infiltration in READ. CDH1 gene arm-level gain or NOS2 gene arm-level deletion only significant influenced the infiltration of dendritic cell in READ, respectively. However, in COAD, multiple immune cells infiltration were significantly inhibited by variety SCNA of CDH1. Besides, NAT2, TIMP1 and TP53 genetic alterations also exhibited the similar situation. These results indicated that the genetic alterations of the 7 prognostic genes could be responsible for the immune escape of CRC, partly. More importantly, COAD patients with the genetic alterations of the 7 prognostic genes about deep deletion, arm-level deletion and gain, maybe insensitive to immunotherapy regimens. However, the efficacy of immunotherapy may not be significantly affected in READ patients with the genetic alterations, or the influence would be far less than that in COAD patients, alternatively.

MSI (Microsatellite instability) and TMB (Tumor mutational burden) are an emerging cancer immunotherapy biomarkers [32]. To further evaluate the sensitivity of prognostic signature to immunotherapy, we subsequently compared the TMB score and MSI score of high-risk group and low-risk group, respectively. The results showed that there was no significant difference in TMB score between the two groups (*p* = 0.62), whereas the MSI score of high-risk group was significantly higher than those of low-risk group (*p* = 0.015) (Fig. 4B).

DNA mismatch repair (MMR) is crucial to ensure the integrity of the genome. Cancer with a defective MMR is a main reason that lead to a higher MSI [33]. Thus, we also analyzed the correlation between prognostic characteristics (Riskscore) and the expression of MMR genes, including MLH1, MSH2, MSH6, and PMS2 whose deficiency being the most common in the MMR deficient tumors[33]. The results showed that these four genes expression were significantly negatively correlated with prognostic characteristics (*p* < 0.05) (Fig. 4C).

### Functions and pathways mediated by the 7 prognostic genes

**Fig. 5:**
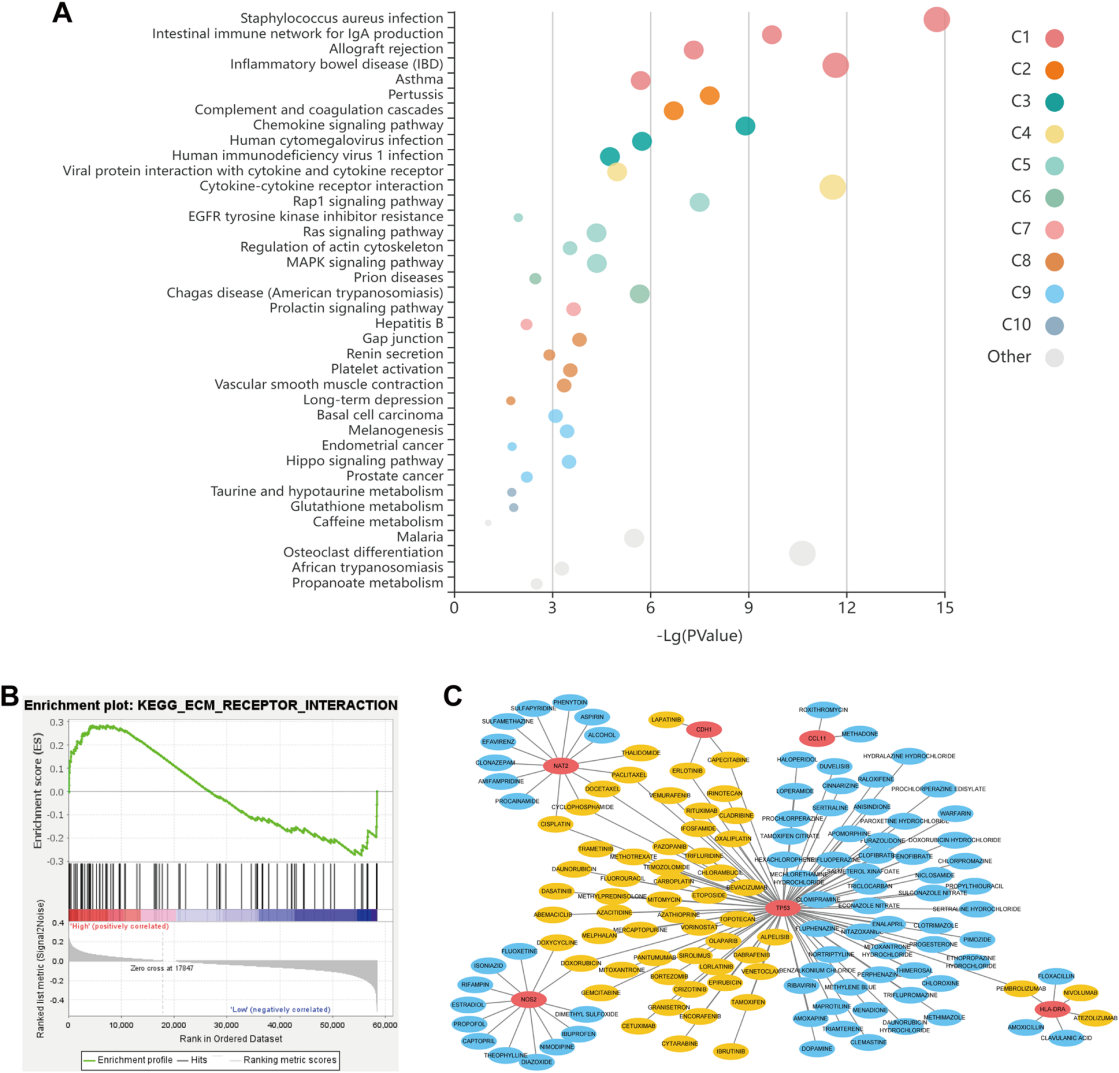
Pathway enrichment analysis and drug–gene interaction. (**A**) Functions of IBDGs and their significantly associated genes were analyzed by KEGG using KOBAS online tools. **B** Significantly enriched pathway in high-risk patients using gene-set enrichment analysis. **C** Drug–gene interaction network. The red nodes represent the 7 prognostic genes. The orange nodes represent approved antineoplastic drugs and the blue nodes represent approved non-antineoplastic drugs

To explore the pathways mediated by the CDH1, CCL11, HLA–DRA, NOS2, NAT2, TIMP1 and TP53, we collected 100 most frequently altered neighbor genes of the 7 prognostic genes in CRC from GEPIA database, and KEGG pathway analysis was performed using KOBAS online tool. The results showed that these genes were participates in chemokine signaling pathway, Rap1 signaling pathway, Ras signaling pathway, MAPK signaling pathway and Hippo signaling pathway (Fig. 5A), which were related to the occurrence and pathogenesis of IBD and CRC.

Meanwhile, in view of the difference in prognosis between high-risk group and low-risk group may be related to the enrichment of some important pathways, we also performed gene set enrichment analysis (GSEA). As the results shown in Fig. 5B, Additional file 1: Figure S6 and Table S2, 6 pathways were significantly enriched in the high-risk group, especially the pathway of extracellular matrix (ECM) receptor interaction which has been reported that is a crucial pathway involved in colorectal cancer progression and metastasis [34].

### *Drug*–*gene interaction network*

Based on the DGIdb database, drugs that target prognostic genes were analyzed to explore the potential anti-colorectal cancer drugs. The result showed that there were 136 approved drugs targeting the 6 prognostic genes, and 58 of which have been approved for tumor treatment (Fig. 5C). In the drugs, except for Capecitabine, Panitumumab, Cetuximab, Irinotecan, Oxaliplatin and Capecitabine which are the well-known anti-colorectal cancer drugs in clinic, the others may also have potential effect of anti-colorectal cancer. However, further experimental verifications are required.

## Discussion

As the world’s top three common cancer, CRC is also a major cause of deaths for cancer. More and more studies reported that CRC is mostly evolved from precancerous lesions. IBD is considered to be a major risk factor of CRC due to its chronic intestinal inflammatory process. CRC patients with IBD history show poorer prognosis and higher mortality rates than sporadic CRC patients [4, 10]. Alterations of IBD-associated genes undoubtedly contribute to colorectal carcinogenesis. This reinforces the importance of finding new biomarkers for early prediction, diagnosis, prognosis, and treatment of IBD-related CRC.

In this study, we focused on 132 IBDGs and analyzed their transcriptional levels in CRC tumors and normal colorectal tissues, primarily. Meanwhile, we also explored the potential reason that cause the differential expression of IBDGs, through analyzing the transcription factor binding sites in the gene promoter regions, separately. We found that the upregulation of the 32 IBDGs might relate to the overexpression of transcription factor Ascl2, KLF16, KLF5, TCF3 and TFDP1 in CRC. Simultaneously, the downregulation of transcription factor EBF1, KLF9, MZF1 and ZEB1 might responsible for the downregulation of 11 IBDGs in CRC. These results indicated that the 9 transcription factors may play important roles in tumorigenic transformation and tumor immune regulation of IBD-related CRC. Regulating these transcription factors may contribute to improve the immunotherapy of CRC, which still faces many challenges in clinic, including target deficiency, drug resistance and toxicity [35, 36].

We further analyzed the effect of IBDGs on the pathological process of CRC patients through GEPIA database and Kaplan–Meier plotter online tool. We found that 13 IBDGs displayed highly variable with the CRC progression, and 49 IBDGs were closely associated with the prognosis of colon and rectal cancer patients. Then, through LASSO Cox regression analysis, we screened out 7 prognostic genes, including CDH1, CCL11, HLA–DRA, NOS2, NAT2, TIMP1 and TP53 from IBDGs which differentially expressed in CRC tumor and normal tissue, and significantly related to pathological stages and prognosis of CRC patients. Based on the 7 prognostic genes and 217 cases CRC patients’ clinical information, the prognostic signature of CRC was established. Meanwhile, the property of the prognostic signature was also verified in test cohort with 216 cases CRC patients, and the results proved that the model had an excellent ability in risk stratification and prognostic prediction.

In recent years, increasing evidence indicates that immune cells infiltration, which is an important factor responsible for the gene expression changes in tumor, plays a key role in anti-tumor immune surveillance and contributes to the prognosis of cancer patients [37]. Meanwhile, genetic alterations also significantly influence the infiltration of immune cells, and closely associated with immunotherapeutic effect of different tumors [38]. In this study, we noticed that CDH1, CCL11, HLA–DRA, NOS2, NAT2 and TIMP1 exhibited significant positive or negative correlation with the infiltration of multiple immune cells in COAD and READ, such as B cells, CD8 + T cells, CD4 + T cells, macrophages, neutrophils or dendritic cells, except for TP53. More importantly, the 7 prognostic genes were altered in 43% CRC patients, especially gene missense mutation, amplification and deep deletion. These genetic alterations significantly inhibited the infiltration of different immune cells in colon cancer especially, including CD8 + T cells which are the key undertakers of anti-tumor immunity [39]. Genomic alterations during tumorigenesis cause the production of antigens on the tumor surface, which recognized by the immune system, triggering the anti-tumor immune response to inhibit tumor progression [40]. Thus, the inhibition of immune cells infiltration of the 7 prognostic genes with genetic alterations could be responsible for the tumor immune escape and poor prognosis of COAD, partly. Interestingly, on the whole, the effect of the 6 genes (No data related to HLA–DRA was retrieved in the TIMER database) with genetic alterations on immune cells infiltration in READ was not obvious, compared with in COAD. These discoveries implied that immunotherapy drugs which are effective in treating COAD with wild-type CDH1, CCL11, NOS2, NAT2, TIMP1 and TP53, maybe insensitive to the patients with the genetic alterations about deep deletion, high amplification, arm-level deletion and gain. What’s more, the efficacy of immunotherapy may not be significantly affected in READ patients with the genetic alterations, or the influence would be far less than that in COAD, alternatively. This study offers an insight in understanding the potential role of the 7 prognostic genes as biomarkers in tumor immunology and individualized drug administration.

Moreover, through ESTIMATE algorithm we found that the immune score of low-risk group was significantly higher than that of high-risk group, suggesting the infiltration of immune cells had a significant difference between the two groups. Indeed, compared with the low-risk group, the immune infiltration levels of activated T cells CD4 memory, neutrophils, dendritic cells resting, B cell naïve, plasma cells, macrophages M1 and resting T cells CD4 memory were significantly down-regulated in the high-risk group. These results suggested that the down-regulation of immune cells infiltration might be responsible for the poor prognosis of the high-risk group, at least partly. Then, in the treatment of CRC, simultaneously targeted intervention of these 7 prognostic genes may help improve the outcome of high-risk patients. Automatically, to further explore the potential therapeutic strategies for CRC, we construct drug–gene interaction network based on the DGIdb database. We found that 136 approved drugs, including 58 which have been approved for tumor treatment, targeting the 6 prognostic genes. In the anti-tumor drugs, 6 drugs are the already widely used anti-colorectal cancer drugs in clinic. According to our current research, the others may also have potential effect of anti-colorectal cancer. Besides, 78 drugs which are used to treat diseases other than cancers perhaps contribute to improving the prognosis of CRC patients.

Pathological studies shown that microsatellite instability(MSI)is associated with increased number of mutations in tumoral DNA which correspond to the increased presence of circulating antitumor lymphocytes and neoantigens [41]. Meanwhile, MSI is closely relate to the tumorigenesis of CRC, and has a significant effect on immunotherapy [42]. Through analyzing clinical data in TCGA database, we found that the MSI score of high-risk group was significantly higher than those of low-risk group. This suggested that the 7 prognostic genes-based risk stratification was related to MSI. Existing theories demonstrated that the main reason of higher MSI is defective mismatch repair (MMR). MMR is an intracellular process that identify and correct base–base mismatches and insertions/deletions (indels) generated during DNA replication and recombination [33]. MMR strictly regulated by MLH1, MSH2, MSH6 and PMS2, and their dysfunction would lead to cancer hypermutated and accumulate mutations in monomorphic microsatellites [43]. Thus, we subsequently analyzed the correlation between prognostic characteristics (Riskscore) and the expression of MMR genes. Consistently, we found that the prognostic characteristics were significantly negatively correlated with MLH1, MSH2, MSH6 and PMS2 expression. These all supported that the 7 prognostic genes could be used as valuable biomarkers for prognostic diagnosis of CRC patients.

Finally, we analyzed the function and pathways mediated by the prognostic genes. We found that 7 prognostic genes and their 100 most frequently altered neighbor genes were enriched in chemokine signaling pathway, Rap1 signaling pathway, Ras signaling pathway, MAPK signaling pathway and Hippo signaling pathway. Noteworthily, the important roles of these signal pathways played in the occurrence and pathogenesis of IBD and CRC have been widely reported. Besides, we also explored the potential pathways that related to the difference in prognosis between high- and low-risk groups. Among the 6 signal pathways obtained by GSEA, the pathway of ECM receptor interaction was attracted our attention for its crucial roles in CRC progression and metastasis [34]. Together, these results emphasized that the chronic inflammatory process of IBD caused the abnormal regulation of the prognostic genes and their related signal pathways enduringly, perhaps the important reason for the tumorigenesis and poor prognosis of IBD related CRC.

Although our results have potential clinical significance, they still have some limitations. Further cell biology experiments and clinical studies are needed to verify the results. Besides, the potential mechanisms, molecules interactions and clinical applications of the prognostic genes in CRC also need to be explored.

## Conclusions

In this study, we systematically analyzed the expression, pathological stages and prognostic value of IBDGs in CRC, and 7 prognostic genes including CDH1, CCL11, HLA–DRA, NOS2, NAT2, TIMP1 and TP53 were screened. Based on the 7 prognostic genes, a prognostic signature was established, and its clinical value in risk stratification and prognostic prediction were also verified. Besides, through immune cells infiltration analysis, we found that the infiltration of immune cells had a significant difference between low-risk group and high-risk group. Meanwhile, the effect of genetic alterations of the prognostic genes on immune cells infiltration also exhibited significant difference between COAD and READ. More importantly, 7 prognostic genes-based risk stratification was associated with MSI, and its prognostic characteristics were significantly negatively correlated with MMR genes. These all supported that the 7 prognostic genes could be used as valuable biomarkers for prognostic diagnosis and personalized immunotherapy of CRC patients.

## Supplementary Information

**Additional file 1: Table S1** Correlation between the 7 prognostic genes and immune cells infiltration. **Table S2 **Significantly enriched pathways in high-risk patients using gene-set enrichment analysis. **Figure S1** (A, C) Transcription factor binding sites analysis in the promoter regions of upregulated (A) and downregulated IBDGs (C) using EPD database. (B, D) The expression of 9 key transcription factors in colorectal cancer were analyzed using GEPIA database (*P < 0.05).** Figure S2. **Prognostic value of IBDGs in rectal cancer patients were evaluated using Kaplan–Meier plotter database.** Figure S3** Establishment and verification of prognostic signature based on IL4R, IL2RB and NAT2. (A, D) The survival status, riskscores and prognostic signature expression levels of each patient in training and test cohort. (B, E) Kaplan–Meier survival curve between high and low riskscore patients in training and test cohort. (C, F) Time-dependent (1–5 years) ROC curve comparison of training and test cohort.** Figure S4** Verification of prognostic signature in entire cohort. (A) The survival status, riskscores and prognostic signature expression levels of each patient in entire cohort. (B) Kaplan–Meier survival curve between high and low riskscore patients in entire cohort. (C) Time-dependent (1–5 years) ROC curve comparison of entire cohort.** Figure S5 **Correlations between somatic copy number alterations (SCNA) of the 7 prognostic genes and immune infiltration level in COAD and READ were analyzed through TIMER database (two-sided Wilcoxon rank sum test. *P < 0.05, **P < 0.01 and ***P < 0.001).

## Data Availability

The original contributions presented in the study are included in the article/ Supplementary Material. Further inquiries can be directed to the corresponding authors.
