# Supplementary material for: Prognostic and tumor immunity implication of inflammatory bowel disease-associated genes in colorectal cancer
Source: Eur J Med Res. 2022 Jun 13;27:91. doi: 10.1186/s40001-022-00720-0 (PMC9190109; doi:10.1186/s40001-022-00720-0)
Supplement: Supplementary file 1 — Additional file 1: Table S1 Correlation between the 7 prognostic genes and immune cells infiltration. Table S2 Significantly enriched pathways in high-risk patients using gene-set enrichment analysis. Figure S1 (A, C) Transcription factor binding sites analysis in the promoter regions of upregulated (A) and downregulated IBDGs (C) using EPD database. (B, D) The expression of 9 key transcription factors in colorectal cancer were analyzed using GEPIA database (*P < 0.05). Figure S2. Prognostic value of IBDGs in rectal cancer patients were evaluated using Kaplan–Meier plotter database. Figure S3 Establishment and verification of prognostic signature based on IL4R, IL2RB and NAT2. (A, D) The survival status, riskscores and prognostic signature expression levels of each patient in training and test cohort. (B, E) Kaplan–Meier survival curve between high and low riskscore patients in training and test cohort. (C, F) Time-dependent (1–5 years) ROC curve comparison of training and test cohort. Figure S4 Verification of prognostic signature in entire cohort. (A) The survival status, riskscores and prognostic signature expression levels of each patient in entire cohort. (B) Kaplan–Meier survival curve between high and low riskscore patients in entire cohort. (C) Time-dependent (1–5 years) ROC curve comparison of entire cohort. Figure S5 Correlations between somatic copy number alterations (SCNA) of the 7 prognostic genes and immune infiltration level in COAD and READ were analyzed through TIMER database (two-sided Wilcoxon rank sum test. *P < 0.05, **P < 0.01 and ***P < 0.001). [file 40001_2022_720_MOESM1_ESM.docx]

**Additional file**

**Prognostic and Tumor Immunity Implication of Inflammatory Bowel Disease-Associated Genes in Colorectal Cancer**

Di Wang^1^, Biao Xie^1^*

^1^ Department of Gastroenterology, People's Hospital of Longhua, Shenzhen 518109, P.R. China

* To whom correspondence should be addressed at Department of Gastroenterology, people's Hospital of Longhua, Shenzhen 518109, P.R. China

Xie B (Tel/Fax: +0755-27741585 email: [xiebiaohxq@163.com](mailto:xiebiaohxq@163.com))

**
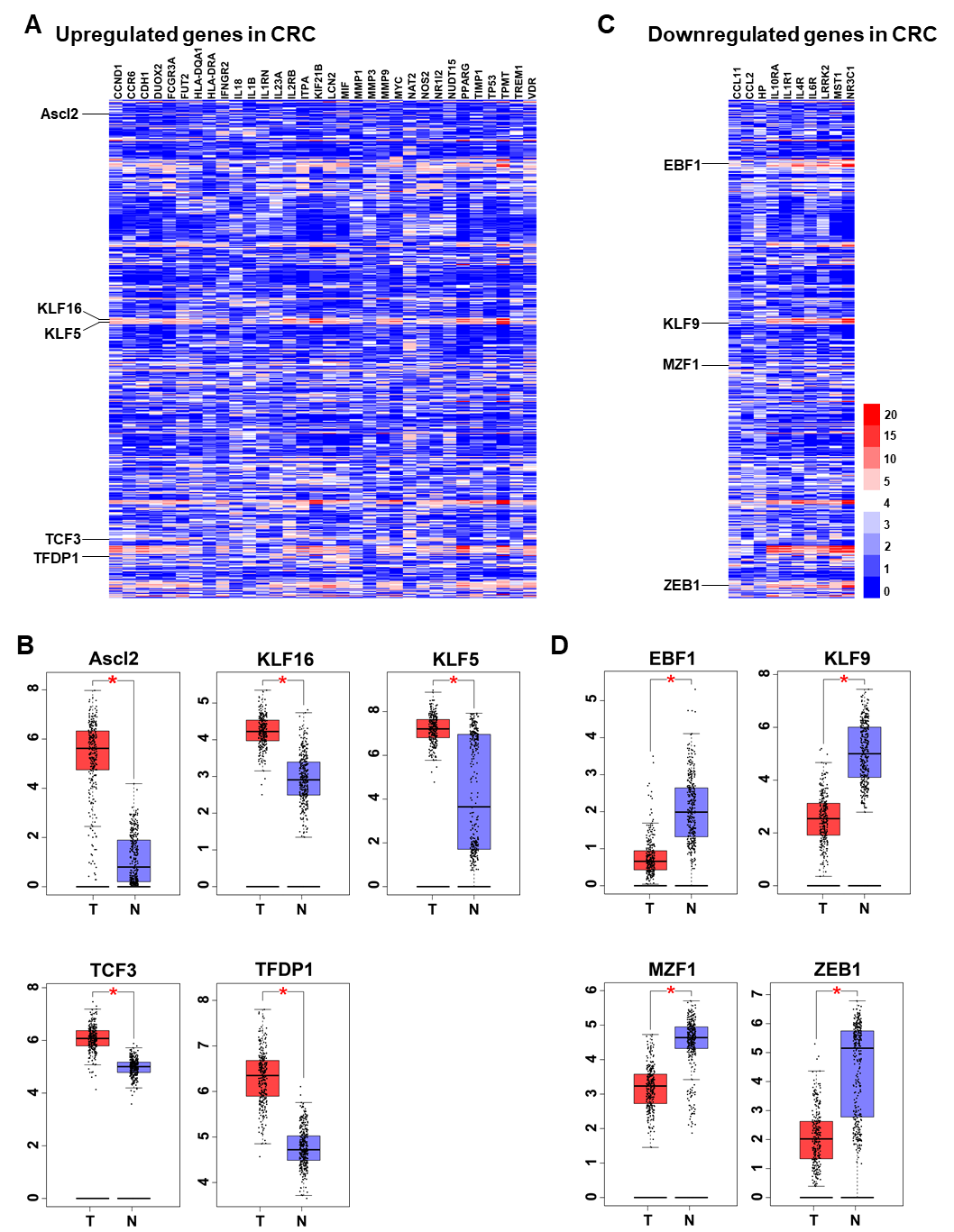
**

**Figure S1.** (A, C) The transcription factor binding sites analysis in the promoter regions of upregulated (A) and downregulated IBDGs (C) using EPD database. (B, D) The expression of 9 key transcription factors in colorectal cancer were analyzed using GEPIA database (**P* < 0.05).

**
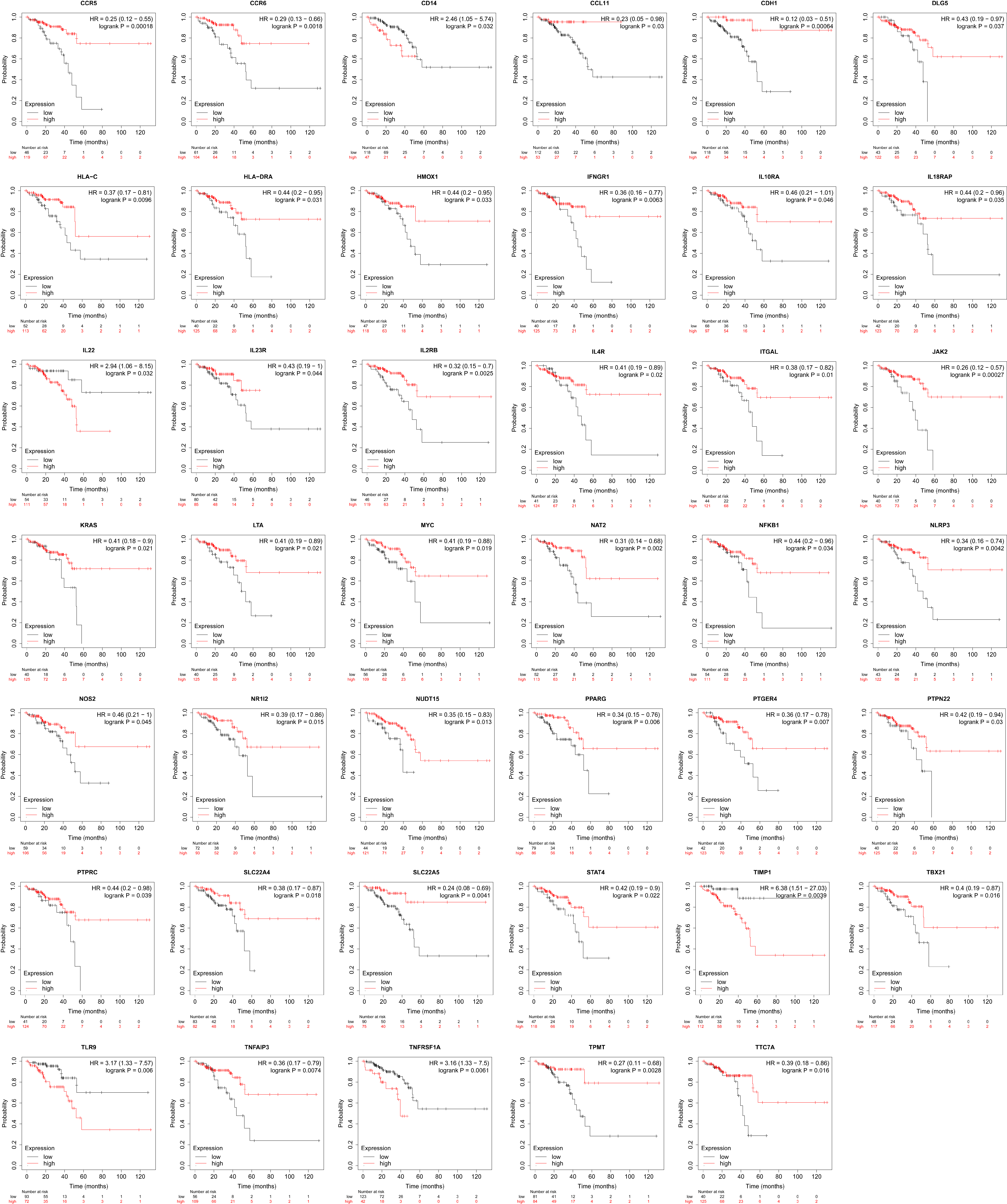
**

**Figure S2.** The prognostic value of IBDGs in rectal cancer patients were evaluated using Kaplan-Meier Plotter database.


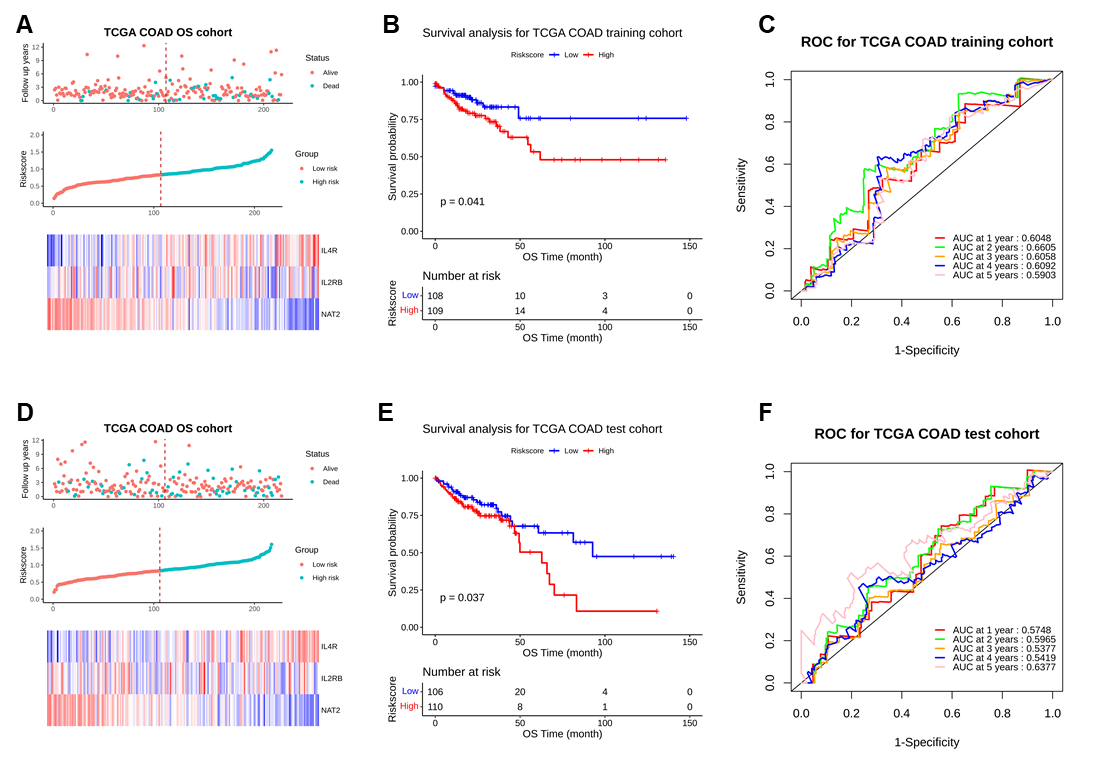


**Figure S3.** The establishment and verification of prognostic signature based on IL4R, IL2RB and NAT2. (A, D) The survival status, riskscores and prognostic signature expression levels of each patient in training and test cohort. (B, E) Kaplan-Meier survival curve between high and low riskscore patients in training and test cohort. (C, F) Time-dependent (1-5 years) ROC curve comparison of training and test cohort.


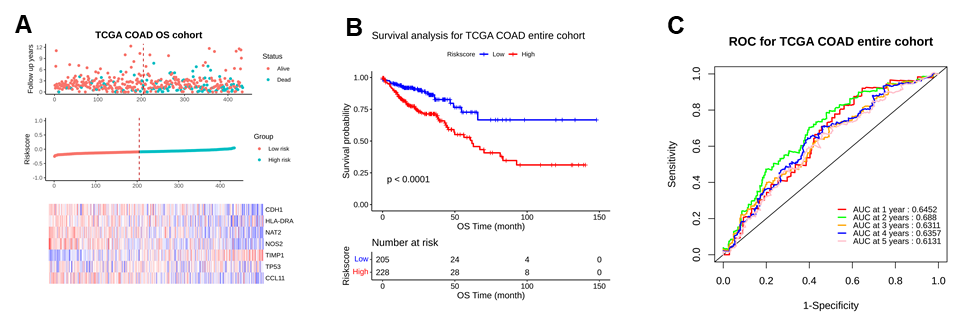


**Figure S4.** The verification of prognostic signature in entire cohort. (A) The survival status, riskscores and prognostic signature expression levels of each patient in entire cohort. (B) Kaplan-Meier survival curve between high and low riskscore patients in entire cohort. (C) Time-dependent (1-5 years) ROC curve comparison of entire cohort.


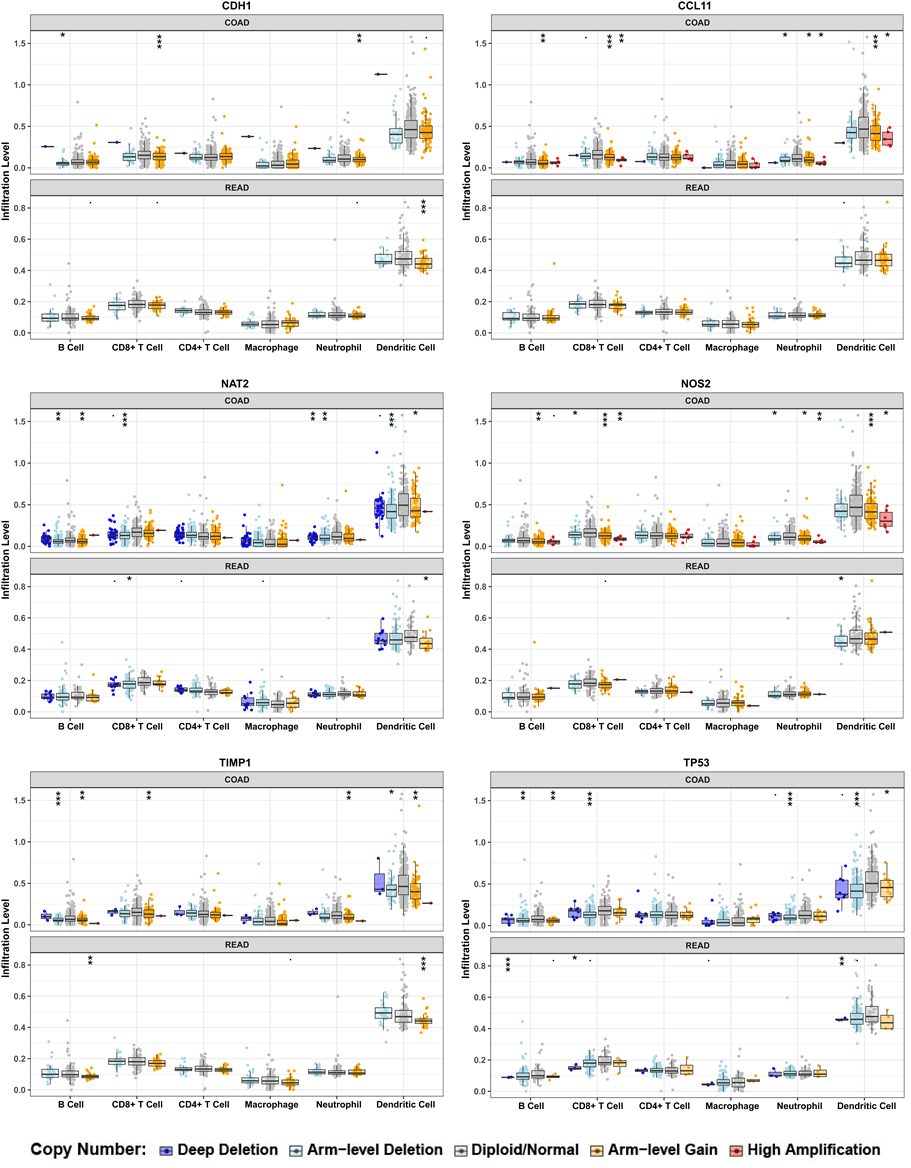


**Figure S5.** The correlations between somatic copy number alterations (SCNA) of the 7 prognostic genes and immune infiltration level in COAD and READwere analyzed through TIMER database (two-sided Wilcoxon rank sum test. **P* < 0.05, ***P* < 0.01 and ****P* < 0.001).


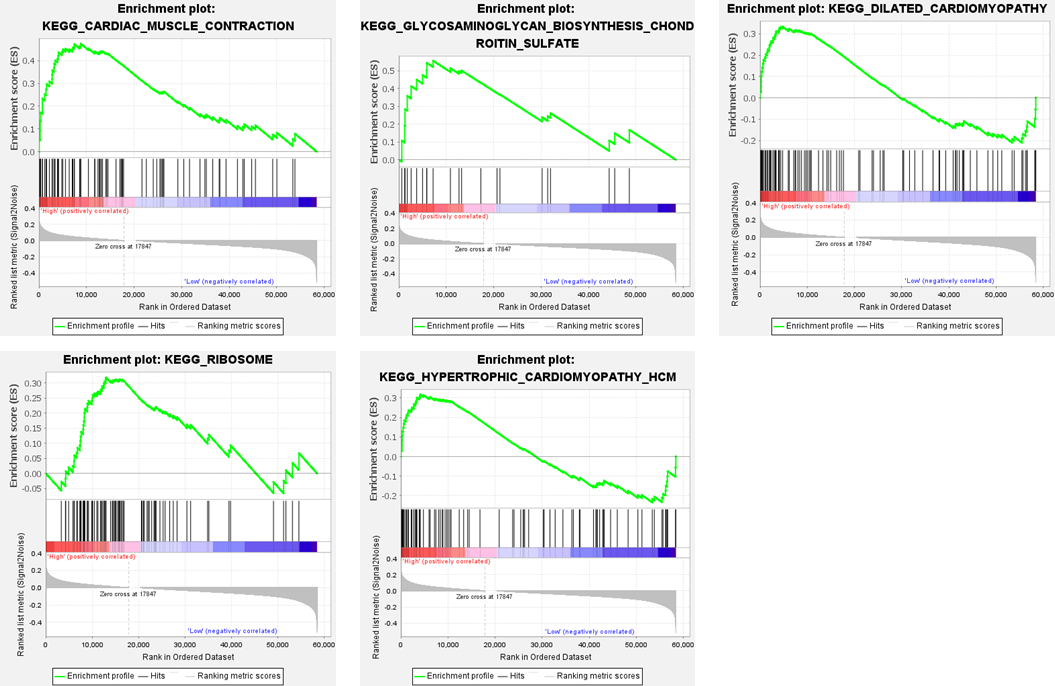


**Figure S6.** Significantly enriched pathways in high-risk patients using gene-set enrichment analysis.

**Table S1** **The correlation between the 7 prognostic genes and immune cells infiltration**

| **COAD** |  |  |  |  |  |  |  |  |  |  |  |  |
| --- | --- | --- | --- | --- | --- | --- | --- | --- | --- | --- | --- | --- |
| Genes | B Cell | | CD8+ T Cell | | CD4+ T Cell | | Macrophage | | Neutrophil | | Dendritic Cell | |
|  | partial.cor | Log(*p*) | partial.cor | Log(*p*) | partial.cor | Log(*p*) | partial.cor | Log(*p*) | partial.cor | Log(*p*) | partial.cor | Log(*p*) |
| CDH1 | 0.15 | -2.60 | 0.10 | -1.28 | 0.32 | -10.19 | 0.21 | -4.55 | 0.08 | -0.98 | 0.15 | -2.69 |
| CCL11 | 0.17 | -3.34 | 0.13 | -2.05 | 0.36 | -12.88 | 0.39 | -15.35 | 0.36 | -13.23 | 0.36 | -12.95 |
| NOS2 | 0.12 | -1.81 | 0.06 | -0.60 | 0.02 | -0.20 | -0.21 | -4.62 | 0.22 | -5.04 | 0.15 | -2.73 |
| HLA-DRA | 0.32 | -10.37 | 0.46 | -21.51 | 0.29 | -8.74 | 0.43 | -18.68 | 0.71 | -62.57 | 0.73 | -66.38 |
| TIMP1 | -0.05 | -0.46 | 0.22 | -5.01 | 0.17 | -3.25 | 0.37 | -13.97 | 0.38 | -14.55 | 0.36 | -12.90 |
| TP53 | 0.01 | -0.10 | 0.01 | -0.04 | -0.05 | -0.53 | -0.06 | -0.70 | -0.05 | -0.44 | 0.02 | -0.17 |
| NAT2 | 0.17 | -3.11 | 0.11 | -1.57 | 0.05 | -0.48 | -0.07 | -0.76 | -0.12 | -1.72 | -0.03 | -0.25 |
|  |  |  |  |  |  |  |  |  |  |  |  |  |
| **READ** |  |  |  |  |  |  |  |  |  |  |  |  |
| Genes | B Cell | | CD8+ T Cell | | CD4+ T Cell | | Macrophage | | Neutrophil | | Dendritic Cell | |
|  | partial.cor | Log(*p*) | partial.cor | Log(*p*) | partial.cor | Log(*p*) | partial.cor | Log(*p*) | partial.cor | Log(*p*) | partial.cor | Log(*p*) |
| CDH1 | 0.21 | -1.83 | 0.33 | -4.09 | -0.02 | -0.10 | 0.23 | -2.13 | 0.18 | -1.51 | 0.01 | -0.05 |
| CCL11 | 0.11 | -0.67 | 0.06 | -0.35 | 0.17 | -1.31 | 0.11 | -0.69 | 0.25 | -2.58 | 0.27 | -2.89 |
| NOS2 | 0.24 | -2.39 | 0.12 | -0.76 | 0.02 | -0.08 | -0.20 | -1.74 | 0.09 | -0.53 | 0.28 | -3.03 |
| HLA-DRA | 0.30 | -3.42 | 0.31 | -3.69 | 0.18 | -1.49 | 0.19 | -1.64 | 0.28 | -3.10 | 0.69 | -20.20 |
| TIMP1 | -0.19 | -1.63 | -0.15 | -1.12 | 0.24 | -2.39 | 0.36 | -4.89 | 0.18 | -1.40 | 0.15 | -1.09 |
| TP53 | 0.10 | -0.66 | -0.05 | -0.23 | -0.06 | -0.32 | -0.04 | -0.17 | -0.09 | -0.56 | -0.13 | -0.86 |
| NAT2 | 0.18 | -1.50 | 0.35 | -4.61 | -0.36 | -4.75 | -0.14 | -0.95 | 0.06 | -0.29 | -0.06 | -0.29 |

**Table S2 Significantly enriched pathways in high-risk patients using gene-set enrichment analysis**

| **Pathway** | **NES** | ***p*** |
| --- | --- | --- |
| KEGG_CARDIAC_MUSCLE_CONTRACTION | 2.3343 | 0.0005 |
| KEGG_GLYCOSAMINOGLYCAN_BIOSYNTHESIS_CHONDROITIN_SULFATE | 2.0079 | 0.0023 |
| KEGG_DILATED_CARDIOMYOPATHY | 1.6889 | 0.0153 |
| KEGG_RIBOSOME | 1.5897 | 0.0250 |
| KEGG_HYPERTROPHIC_CARDIOMYOPATHY_HCM | 1.5638 | 0.0233 |
| KEGG_ECM_RECEPTOR_INTERACTION | 1.3986 | 0.0479 |
